# Exon-array profiling unlocks clinically and biologically relevant gene signatures from formalin-fixed paraffin-embedded tumour samples

**DOI:** 10.1038/bjc.2011.66

**Published:** 2011-03-15

**Authors:** J S Hall, H S Leong, L S C Armenoult, G E Newton, H R Valentine, J J Irlam, C Möller-Levet, K A Sikand, S D Pepper, C J Miller, C M L West

**Affiliations:** 1 Translational Radiobiology Group, School of Cancer and Enabling Sciences, Manchester Academic Health Science Centre, The University of Manchester, Wilmslow Road, Manchester M20 4BX, UK; 2 Applied Computational Biology and Bioinformatics Group. The Paterson Institute for Cancer Research, Wilmslow Road, Manchester M20 4BX, UK; 3 Molecular Biology Core Facility, The Paterson Institute for Cancer Research, Wilmslow Road, Manchester M20 4BX, UK; 4 The Christie Hospital NHS Foundation Trust; Wilmslow Road, Manchester M20 4BX, UK

**Keywords:** cervix cancer, exon array, expression profiling, FFPE, histology

## Abstract

**Background:**

Degradation and chemical modification of RNA in formalin-fixed paraffin-embedded (FFPE) samples hamper their use in expression profiling studies. This study aimed to show that useful information can be obtained by Exon-array profiling archival FFPE tumour samples.

**Methods:**

Nineteen cervical squamous cell carcinoma (SCC) and 9 adenocarcinoma (AC) FFPE samples (10–16-year-old) were profiled using Affymetrix Exon arrays. The gene signature derived was tested on a fresh-frozen non-small cell lung cancer (NSCLC) series. Exploration of biological networks involved gene set enrichment analysis (GSEA). Differential gene expression was confirmed using Quantigene, a multiplex bead-based alternative to qRT–PCR.

**Results:**

In all, 1062 genes were higher in SCC vs AC, and 155 genes higher in AC. The 1217-gene signature correctly separated 58 NSCLC into SCC and AC. A gene network centered on hepatic nuclear factor and GATA6 was identified in AC, suggesting a role in glandular cell differentiation of the cervix. Quantigene analysis of the top 26 differentially expressed genes correctly partitioned cervix samples as SCC or AC.

**Conclusion:**

FFPE samples can be profiled using Exon arrays to derive gene expression signatures that are sufficiently robust to be applied to independent data sets, identify novel biology and design assays for independent platform validation.

Archival formalin-fixed paraffin-embedded (FFPE) samples contain a wealth of information pertaining to clinical disease, particularly cancer. Although FFPE is the standard tissue preservation method worldwide, constituent nucleic acids suffer from chemical modification and degradation during sample processing and storage (Lee *et al*, 2005), rendering archival RNA incompatible with most high-throughput molecular techniques. Microarray technology allows high-throughput RNA expression analysis and predictive signature generation, such as the use of the Oncotype DX array to profile node-negative, oestrogen receptor positive breast cancers (Paik *et al*, 2004). The existence of substantial libraries of archival material with long-term clinical data makes their application to FFPE samples particularly appealing. However, issues with RNA quality mean that failure rates as high as 76% have been reported (Penland *et al*, 2007). Significant effort has been made to modify existing microarray protocols to enhance their compatibility with FFPE material (Linton *et al*, 2009; Abdueva *et al*, 2010), and good concordance between FFPE and fresh-frozen material (>90% overlap in detected probesets) is now achievable (Farragher *et al*, 2008; Linton *et al*, 2009). Despite these advances, there is reluctance to work with or trust FFPE data, and the vast repository of archival samples with precious long-term follow-up remains largely untapped, in favour of prospectively collected material, prepared and stored at great expense. As microRNAs are well preserved in formalin, there is interest in searching for microRNA biomarkers in routinely processed material (Lebanony *et al*, 2009). However, as the majority of microRNAs are poorly characterised, this approach disregards the wealth of data regarding gene function.

Here, we investigate the feasibility of gene expression signature generation from archival FFPE tissues. We describe an ensemble of recently developed molecular and computational techniques, which when used in concert unlock clinically and biologically meaningful gene information from FFPE samples. In particular, we make use of Affymetrix Exon 1.0 ST arrays, which were originally designed to study alternative splicing. These arrays feature probesets distributed along the length of each gene, rather than simply targeting the 3′ end, as is done with conventional 3′ IVT arrays. This intrinsic redundancy offers potential advantages with FFPE material, as it increases the likelihood of detecting intact and measurable RNA for each gene of interest. Furthermore, additional confidence can be attributed to genes that have multiple, differentially expressed and concordant probesets. As the arrays require different RNA preparation protocols and analysis to ‘standard’ Affymetrix arrays, the study serves to validate this novel use of the arrays themselves, the upstream protocols used to prepare material for hybridisation, and the downstream computational biology tools used for their analysis.

We, like many groups around the world are interested in generating gene expression signatures relating to clinical parameters, such as survival, specifically in cervix cancer. However, before carrying out this signature generation, we need to ensure our methods are compatible with archival FFPE samples. To allow a clear validation without the requirement for paired fresh-frozen tissues, we asked a question where the answer would be categorically black or white. We hypothesised that the morphogenetic difference between squamous cell carcinoma (SCC) and adenocarcinoma (AC) of the cervix would allow us to be certain of both whether and how well the FFPE methods worked. A further advantage of using this combination is that the protein marker p63 can be used to confirm tumours of squamous origin. (McCluggage, 2007). The principal aim of this study was, therefore, to investigate whether the intrinsic redundancy of Exon arrays could be exploited to derive a gene signature using archival FFPE tumour material.

## Materials and methods

### Patients

FFPE blocks for 28 patients with cervical carcinoma (Supplementary Table S1) were identified from a database of archived specimens, based on histology information from the original pathology report. Squamous cell carcinoma samples (*n*=19) were selected at random, along with all available AC blocks (*n*=9). All tumours contained >30% tumour material. Local ethical approval was obtained for using the human material (LREC: 08/H1011/63).

### RNA extraction and Exon-array hybridisation

RNA was extracted and DNase treated using RecoverAll Total Nucleic Acid Isolation Kit (Ambion, Austin, TX, USA), as per manufacturer's instructions. RNA was quantified using a Bioanalyser (Agilent Technologies Ltd, Santa Clara, CA, USA) and 100 ng amplified using NuGEN WT-Ovation FFPE v2 kit (NuGEN Inc., San Carlos, CA, USA). The WT-Ovation Exon Module V1.0 (NuGEN Inc.) was used to generate ST-cDNA and 4 *μ*g was hybridised to Human Exon 1.0 ST (Affymetrix, Santa Clara, CA, USA) arrays. Further details and raw data (CEL files) are available at http://bioinformatics.picr.man.ac.uk/vice (or GSE27388).

### Microarray data analysis

The microarray data were normalised using RMA (Irizarry *et al*, 2003). The R/BioConductor package xmapcore and the X:Map database (Yates *et al*, 2008) were used to filter non-exonic and multi-targeting probesets. Only probesets that were flagged as ‘present’ (i.e., Detection Above Background (DABG) *P*<0.01) in at least three samples were retained for further analysis. LIMMA (Smyth, 2004) was used to identify probesets that were differentially expressed between SCC and AC subtypes, using FDR <0.01 and absolute fold-change>2 as cutoff. Annotation of differentially expressed genes was performed using GSA (Efron and Tibshirani, 2007), Ingenuity (IPA; Ingenuity Systems) and PAKORA (Leong and Kipling, 2009). The complete bioinformatics analysis pipeline is detailed in Supplementary Methods.

### Validation of SCC and AC gene signature in an independent data set

The microarray results were validated in an independent data set derived from fresh-frozen human non-small cell lung cancer (NSCLC) with known histology (downloaded from GEO database, accession number: GSE10245) (Kuner *et al*, 2009). Affymetrix HG-U133 Plus 2.0 array (Plus 2.0) probesets corresponding to the differentially expressed Exon 1.0 ST probesets were identified using a cross-platform probeset conversion strategy detailed in Supplementary Methods. Mapped probesets were used for supervised clustering analysis.

### QuantiGene

QuantiGene 2.0 Plex Magnetic Separation Assay kit was used (Affymetrix). Probes were designed to specific gene regions anchored by Exon 1.0 ST probesets differentially expressed between SCC and AC. The assay was performed according to the manufacturer's instructions. In triplicate, 150 ng of RNA was added to separate wells of a 96-well hybridisation plate containing magnetic capture beads and QG_2.0 probesets. The plate was incubated for ∼20 h at 55°C and agitated at 600 r.p.m. to maintain suspension. Beads were sequentially hybridised (1 h, 50°C) with Pre-Amplifier probe, the Amplifier probe, the label probe and SAPE with washes in between followed by a final 30 min incubation at 37°C. Bead discrimination and signal detection were performed on a Luminex instrument (Bio-Rad Laboratories, Hercules, CA, USA). Data were exported to Excel, where background subtraction and normalisation to four housekeepers (TBP, GAPDH, PGK1 and YWHAZ) were performed. Quote ‘Panel number 11658’ when specifying QuantiGene probes.

## Results

### Quality control

Twenty-eight FFPE samples were profiled. Independent pathologist review (KAS) confirmed 19 were SCC and 9 AC. Patient demographics and age of the FFPE blocks are summarised in Supplementary Table S1. The blocks had been stored an average of 12 (range 10–16) years. Assessment of over 100 FFPE samples on Affymetrix Exon 1.0 ST arrays showed neither RNA integrity, 260/280, 260/230, concentration nor ST-cDNA yield correlates with percent DABG, a measure of Exon array quality control (data not shown). The only factor found to correlate was sample age, highlighting the importance of comparing material of similar age. In this study, at least 100 ng of total RNA was extracted from all 28 samples, sufficient for NuGen amplification. Similarly, the amplification yield for all samples was >3.8 *μ*g ST-cDNA, sufficient for hybridising to Exon arrays. Post-hybridisation quality control analysis using standard metrics, including percent DABG, did not identify any inconsistent samples, and all samples were included in subsequent analyses.

**Figure 1:**
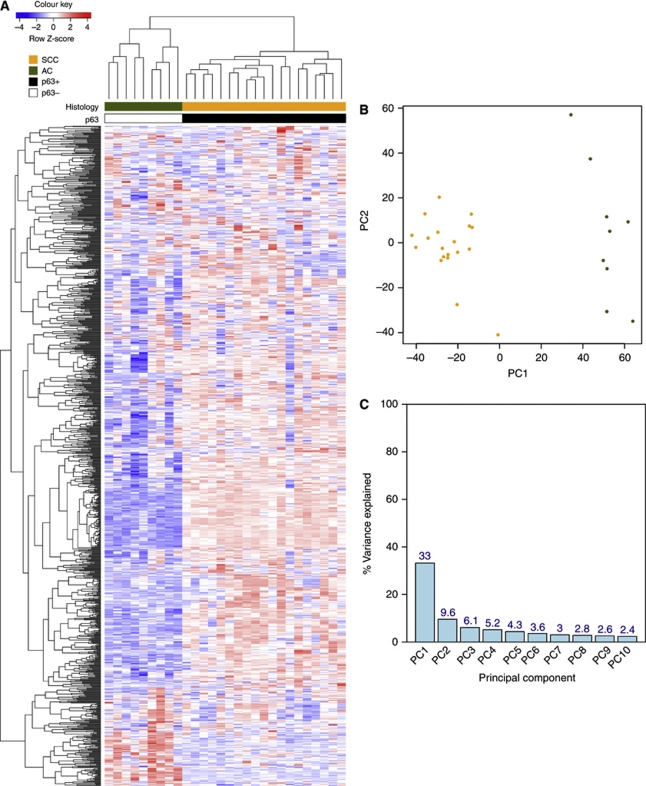
Unsupervised clustering of 1000 probesets with the highest variance across the 28 FFPE cervical cancer samples. (**A**) Each row represents a probeset; each column represents a sample. The expression level of each probeset was standardised by subtracting that probeset's mean expression from its expression value and then dividing by the standard deviation across all the samples. This scaled expression value, denoted as the row Z-score, is plotted in red–blue colour scale with red indicating high expression and blue indicating low expression. Hierarchical clustering of genes and samples was based on Pearson's correlation. The first colour bar at the top indicates the histological classifications of the samples: SCC in orange and AC in green. p63 staining status is indicated by the second coloured bar, where black represents p63+ and white represents p63−. (**B**) Principal component analysis of the top 1000 most variable probesets. Different colours are used for the two histological subtypes: orange represents SCC samples; green represent AC samples. (**C**) Percentage variance explained by the first 10 principal components.

To assess the main source of variation in the experiment, we applied unsupervised hierarchical clustering and principal component analysis, using the 1000 probesets with greatest variance across all 28 samples (Figure 1). In both cases, samples separated according to histological subtypes. Thus, biology is the predominant signal in the data and not that of degradation, sample preparation or processing.

### Molecular stratification of cervical cancer subtypes

Differential gene expression analysis using LIMMA (Smyth, 2004) identified 2673 differentially expressed probesets between SCC and AC (FDR <0.01; absolute fold-change >2). Mapping probesets to genes using xmapcore and X:Map (Yates *et al*, 2008) yielded 1217 non-redundant genes: 1062 expressed higher in SCC than AC; 155 genes higher in AC. The top 30 genes in each group are listed in Supplementary Table S2.

### p63 protein level validation, probe multiplicity and putative marker discovery

**Figure 2:**
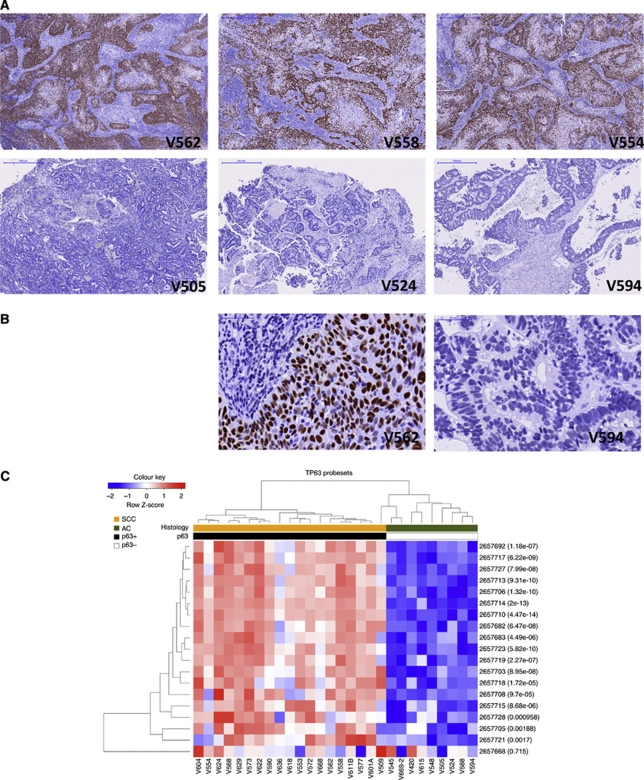
Concordance of TP63 probeset with p63 protein immunohistochemistry. (**A**) SCC (V562, V558 and V554) and AC (V505, V524 and V594) tumours, stained for p63 by immunohistochemistry ( × 5). Brown indicates p63 and blue indicates haematoxylin nuclear counterstain. (**B**) High magnification ( × 20) example of V592 and V594 demonstrating the specificity of the nuclear stain. (**C**) Cluster dendrogram of the 19 TP63 probesets detected above background (DABG *P*<0.01) in our SCC and AC samples. Clustered by samples and by probesets. The scaled expression of each probeset, denoted as the row *Z*-score, is plotted in red–blue colour scale with red indicating high expression and blue indicating low expression. LIMMA *P*-value for each probeset is displayed in brackets. p63 IHC result for each sample is indicated by the p63 bar, coloured black for positive and white for negative.

**Figure 3:**
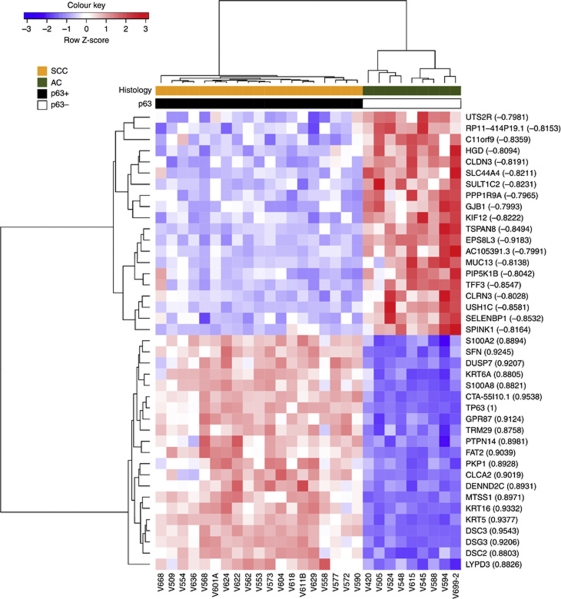
Identification of novel markers of SCC and AC based on TP63 transcript correlation. Hierarchical clustering of genes and samples was based on Pearson's correlation.

The squamous histology marker p63 (McCluggage, 2007) was detected in all 19 SCC, but none of the 9 AC (Figures 2A and B). Twenty-six separate probesets target TP63 on the arrays. Nineteen of the 26 probesets were detected (DABG *P*<0.05; Figure 2C) and 18 were differentially expressed between AC and SCC (FDR <0.01; absolute fold-change >2); only 1, ‘2657668’, was not. The complete agreement with the immunohistochemistry data shows that Exon array measurements of FFPE RNA reflect genuine changes at the protein level. Given these data we compared genes which correlated and anti-correlated with TP63 gene expression, as a method for identifying putative novel markers of SCC and AC (Figure 3). TP63 correlating genes included well-known markers, such as KRT5 (*R*^2^=0.93), along with novel genes such as CTA-55I10.1 (*R*^2^=0.95). TP63 anti-correlated genes included MUC13 (*R*^2^=−0.81) and EPS8L3 (*R*^2^=−0.92).

### Literature-based biological validation

We assessed the expression of four genes known to be specifically expressed in SCC (TP63, DSG3, DSC3 and KRT5) or AC (MUC13, MUC5B, MUC3A and TFF3) (Contag *et al*, 2004; Chao *et al*, 2006; McCluggage, 2007). All eight genes were differentially expressed in the Exon array data and behaved as expected (Supplementary Figure S1A). In addition, the microRNA hsa-miR-205, a newly identified marker of SCC (Lebanony *et al*, 2009), was also found to be differentially expressed between SCC and AC (Supplementary Figure S1B). To assess more robustly the literature, text-based over-representation analysis was performed using PAKORA (Leong & Kipling, 2009). This yielded biologically meaningful terms associated with the different histological subtypes (Supplementary Table S3). Ingenuity Biological Function analysis identified categories such as ‘cancer’ and ‘reproductive system disease’ as expected (Supplementary Table S4). Interestingly, the top canonical pathways associated with SCC-associated genes were ‘Breast Cancer Regulation by Stathmin1’ and ‘HER-2 Signalling in Breast Cancer’. Conversely, the most significant Canonical Pathway associated with AC genes was ‘Maturity Onset Diabetes of Young’ (MODY Signalling, *P*=0.00071).

### Transcription factor binding site and biological network analysis

**Figure 4:**
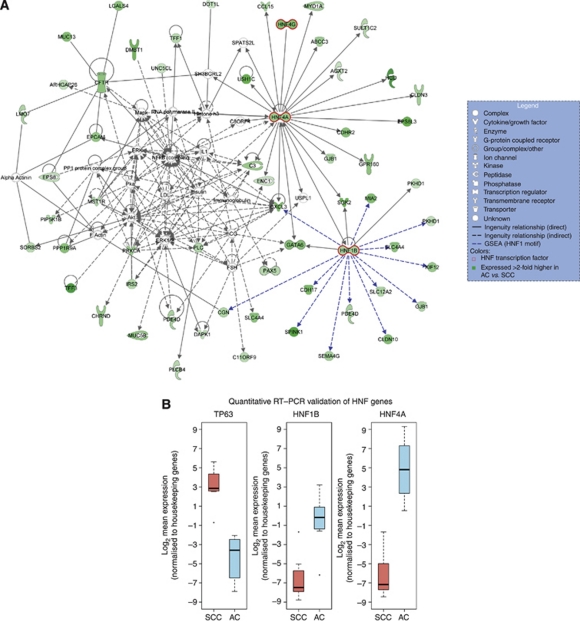
Biological network analysis identifies HNF regulated transcription in adenocarcinoma. (**A**) Network analysis identifies a putative developmental axis centered on hepatic nuclear factor transcription. (**B**) qRT–PCR validation of HNF genes. Data shown are derived from 7 SCC and 6 AC samples.

Gene Set Enrichment Analysis was used to investigate the transcriptional regulation of cellular fate, that is, SCC or AC, focusing on transcription factor binding motifs (GSA (Efron and Tibshirani, 2007); Supplementary Table S5). Four degenerate motifs for hepatic nuclear factor 1 (HNF1) were enriched in the promoter regions of 38 genes associated with AC. Genes with predicted HNF1 transcription factor binding motifs include SPINK1, HNF4A and GATA6. HNF4A and HNF1B were also identified in the MODY signalling pathway, further reinforcing their significance. Figure 4 shows the highest scoring network (score: 100) inferred from the AC gene list using Ingenuity Network Analysis. In addition to the literature links automatically created by Ingenuity (black lines), we manually added genes that have a putative upstream HNF1 motif and were differentially expressed (blue lines). Taken together, these data suggest a role for these transcription factors (GATA6, HNF1B, HNF4A and HNF4G) in AC.

### Cross-validation of the AC/SCC gene signature in an independent fresh-frozen NSCLC cohort

**Figure 5:**
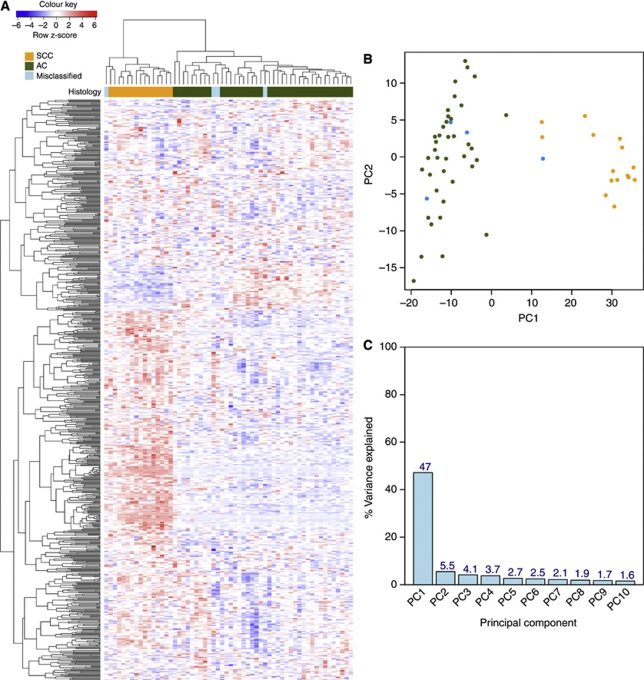
Cross-validation of the FFPE-derived gene signature on an independent fresh-frozen NSCLC data set. (**A**) Hierarchical clustering of genes and samples was based on Pearson's correlation. The scaled expression of each probeset, denoted as the row *Z*-score, is plotted in red–blue colour scale with red indicating high expression and blue indicating low expression. The coloured bar above the heatmap indicates the histological classification: orange=SCC; green=AC; blue=misclassified samples. (**B**) Principal component analysis of the SCC/AC gene signature when applied to the NSCLC data set. The numbers represent the patient IDs. Different colours are used to represent the different histological subtypes: orange=SCC; green=AC; blue=misclassified samples. (**C**) Percentage variance explained by the first 10 principal components.

An independent carcinoma of the cervix data set with histology data was not available publically. A search for any clinical cancer cohort that contained the histological groups SCC and AC identified one data set. This independent cohort comprised 58 fresh-frozen human NSCLC tissue samples hybridised to Plus 2.0 arrays (Kuner *et al*, 2009) (GEO accession: GSE10245). The use of a different tumour type represents a more stringent test of the signature derived in FFPE than if a cervix carcinoma validation cohort was available. Of our 2673 probesets, only 27% had corresponding probesets on the Plus 2.0 array through mapping to common exonic locus, such that the Plus 2.0 array probesets identified are contained within or overlapping the genomic regions spanned by the Exon array probes (see Supplementary Methods for more details). We used xmapcore (Yates *et al*, 2008) to perform this cross-platform probeset conversion and identified 730 Plus 2.0 probesets that targeted 333 genes in the SCC/AC gene signature. When tested on the NSCLC data set, the signature stratified the NSCLC sample as SCC or AC in good agreement with histopathological data (Figure 5). Disagreements were observed for four samples: Pat 342 was classified as AC but clustered to the SCC group, while Pat 30, 55, 188 which were classified as SCC by histology, clustered with the other AC samples. The same discrepancies were apparent in the original study, attributed, by the authors, to sample misclassification (Kuner *et al*, 2009). Thus, the FFPE signature is not only robust, but generalises across cancer types.

### Jackknife analysis to assess stability of the gene signature

**Figure 6:**
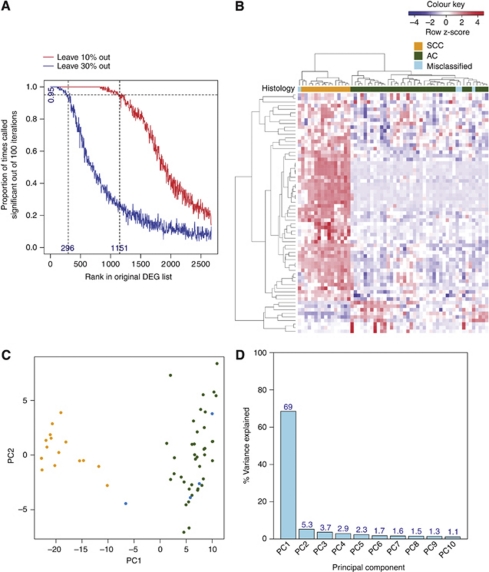
A refined gene signature accurately partitions the NSCLC samples into histological subtypes. (**A**) Proportion of times the 2673 differentially expressed probesets appear as significant in 100 jackknifed data sets (with 10% and 30% of the samples removed) against rank in the original data set. (**B**) Hierarchical clustering of the 296 stable probesets on the 58 NSCLC samples. Genes and samples were clustered based on Pearson's correlation. The scaled expression of each probeset, denoted as the row *Z*-score, is plotted in red–blue colour scale with red indicating high expression and blue indicating low expression. The coloured bar above the heatmap indicates the histological classification: orange=SCC; green=AC; blue=misclassified samples. (**C**) Principal component analysis of the 296 stable probesets when applied to the NSCLC data set. The numbers represent the patient IDs. Different colours are used to represent the different histological subtypes: orange=SCC; green=AC; blue=misclassified samples. (**D**) Percentage variance explained by the first 10 principal components.

Because of transcript fragmentation, there is scepticism over the reproducibility and clinical applicability of results generated from expression profiling FFPE samples. To address this, we assessed the stability of the list of 2673 differentially expressed probesets using jackknife analysis. We removed 10 or 30% of the samples from the original data set, and generated 100 jackknifed data sets for each subsample. The jackknifed data sets were analysed with LIMMA as before to determine how the perturbation affects the composition of the resulting gene lists. Removal of 10% of the samples modified differentially expressed probesets only moderately; with 1151 (43% of original DE probesets) probesets remained significant 95% of the time (Figure 6A). Omitting 30% of samples led to the number of significant probesets declining markedly to 296 (11% of original DE probesets). However, the resulting 296 probesets that were called significant 95% of the time were still sufficiently robust to discriminate between SCC and AC in the independent NSCLC data set (Figures 6B–D).

### Application of a subset of genes using an alternate platform (QuantiGene)

**Figure 7:**
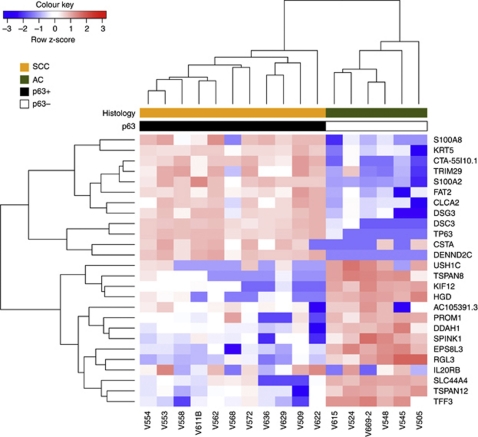
Independent validation of the FFPE signature using an alternative platform. A subset of genes from the cervix FFPE signature was validated by QuantiGene 2.0 Plex. The scaled expression of each probeset, denoted as the row *Z*-score, is plotted in red–blue colour scale with red indicating high expression and blue indicating low expression. Hierarchical clustering of genes and samples was based on Euclidean distance.

We then selected a subset of genes for further study using the QuantiGene 2.0 Plex assay. The 13 most differentially expressed genes in SCC or AC identified by LIMMA analysis were tested across a subset of 11 SCC and 6 AC. There was insufficient RNA for further study for the remaining 11 samples. Unsupervised hierarchical clustering of the resultant data completely separated the cervix tumours into the two histological types (Figure 7). Expression of eight of these genes was also successfully confirmed by qRT–PCR (Supplementary Figure S2). Thus, gene expression changes identified using Exon array profiling of FFPE tumours can be successfully reproduced using alternative technologies.

## Discussion

Recent articles have shown concordance between fresh-frozen and FFPE profiles, using a variety of array technologies (Farragher *et al*, 2008; Linton *et al*, 2009; Di Cesare *et al*, 2010; Saleh *et al*, 2010; Williams *et al*, 2010). While signatures derived from fresh-frozen material have been subsequently tested in FFPE tissue (Roberts *et al*, 2009; Rodriguez *et al*, 2010), this is, to the best of our knowledge, the first time a genome-wide signature has been generated *de novo* from FFPE material and has been shown to successfully stratify an independent clinical data set.

We investigated whether ∼12-year-old FFPE material can be used to derive clinically relevant signatures and discover novel biology. We used standard methods and, in essence, treated our samples and data in a similar way to fresh-frozen material, albeit with a newly developed RNA amplification approach, and an analysis strategy that exploited the intrinsic redundancy of Affymetrix Exon 1.0 ST arrays. The advantage of our pipeline is that the methods are readily implementable using the same array facilities that are already exploited for standard gene expression analyses, with little additional cost.

In cervix cancer, p63 is arguably the best marker of squamous cells (McCluggage, 2007). There is some debate as to its utility in distinguishing between SCC and AC in NSCLC (Au *et al*, 2004) or even whether IHC is a sufficiently robust method for any biomarker (Wolff *et al*, 2007; Hellberg *et al*, 2009). Currently, in a diagnostic setting ‘no marker is totally specific or sensitive for any given lesion’ (McCluggage, 2007). Other biomarkers, which may be more specific, have therefore been pursued, including the microRNA hsa-miR-205 (Lebanony *et al*, 2009). hsa-miR-205 is specifically expressed in SCC compared with AC, and this is supported by the current study, which found the precursor RNA to be over-expressed in SCC (Supplementary Figure S1B). Our identification of a number of genes that correlated or anti-correlated with both TP63 (Figure 3) and hsa-miR-205 (Supplementary Figure S3) suggests they are strong candidates for further study as novel histological markers.

Exon arrays with their increased probe density also have better genomic coverage, and include many newly annotated genes. Along with well-annotated genes that were differentially expressed in our analysis, we also identified a number of genes with no ascribed function. For example, the SCC-specific transcript CTA-55I10.1 is a processed transcript that has no predicted protein coding potential. CTA-55I10.1 is located on chromosome 1 and the 3′-end of this gene overlaps with the microRNA and SCC marker gene hsa-miR-205. Moreover, CTA-55I10.1 shows a similar expression profile to TP63 in the Exon array data (Figure 3) and also in cervix cancer cell line mRNA, with intact RNA (Supplementary Figure S4).

The analysis revealed that SCC genes are predominantly associated with two pathways previously described in breast cancer: Her2 and Stathmin1. Stathmins are over-expressed in many human malignancies (Singer *et al*, 2009), have roles in microtubule depolymerisation, which affect cell motility and viability (Cassimeris, 2002) and as such, are promising therapeutic targets. Furthermore, in AC a network of genes centered on HNF transcription factors (HNF1B, HNF4A and HNF4G) and GATA6 was also identified. It is likely that this HNF–GATA developmental axis has a role in glandular cell differentiation of the cervix, through the transcriptional regulation of multiple target genes (Figure 4A).

Perhaps the strongest validation of our methods is the application of the FFPE-derived signature to an independent clinical data set. Not only did this involve a comparison across array platforms (from Exon 1.0 ST to Plus 2.0 arrays), but also the successful stratification of lung cancer samples using a signature derived from cervix, demonstrating a significant level of robustness to the data. Jackknife analysis further supports this by confirming the overall stability of the results (Figure 6). The resultant 296 probesets, whose differential expression between SCC and AC shows the most stability, are robust candidates for signature validation. Moreover, it was possible to use the Exon array data to design assays using the alternate QuantiGene platform, which recapitulated the original data and stratified cervix samples into histological categories, as expected.

Although the derivation of a classifier suitable for use as a clinical diagnostic is beyond the scope of this publication, the high degree of correspondence found when validating the signature using both qRT–PCR and QuantiGene demonstrates the potential of FFPE in designing targeted assays for clinical exploitation. We feel therefore that these data/methods could be used to this end, and a reduced signature/classifier may be more robust than a single biomarker such as p63 or hsa-miR-205 (Feng *et al*, 2004; Winter *et al*, 2007; Buffa *et al*, 2010).

It has been suggested that expression profiling and biomarker discovery have failed to deliver the much anticipated era of personalised medicine, through the introduction of clinically applicable tests (Diamandis, 2010). While there are many reasons this may be the case, one clear caveat in many studies is the lack of sufficient power to account for biological variation. This along with a paucity of independent data sets to allow refinement of gene signatures has resulted in the bias towards prevalent cancers, such as breast, where prospective studies have resulted in diagnostics reaching the clinic (Paik *et al*, 2004). Prospective collection of samples is costly and requires considerable time and effort to accrue sufficient numbers to address clinically meaningful questions. By utilising the wealth of samples already collected and preserved in FFPE, we overcome sample limitation and facilitate the design and execution of better and more abundant studies in many more centres. Around the world there are an estimated one billion tissue samples archived in hospitals and tissue banks, most of them FFPE (Blow, 2007). This study shows that biological and clinically relevant information can be unlocked from FFPE tumours using whole-genome approaches. The redundancy offered by Exon arrays, which feature multiple probesets targeting individual exons along the length of each gene provide a robust platform for dealing with the uncertainties of RNA extracted from FFPE material. The congruent nature of the results from the different analysis methods (LIMMA, PAKORA, Ingenuity and GSEA) is indicative of high data quality. This is reinforced by the fact that the gene set derived from FFPE cervix tissues performs impressively when applied to an independent fresh-frozen lung cancer data set. While our study focused on the difference between the histological subtypes of cervical SCC and AC to allow us to unambiguously validate our methods, it is reasonable to expect the methods are applicable to many routinely collected FFPE sample sets (e.g., survival series, unknown primaries and so on).

Although working with FFPE material is perhaps not as straightforward as prospectively collected RNA, it is worth noting that sample quality and RNA degradation are not simply restricted to formalin fixation. While international efforts are still required to improve certain areas pertaining to FFPE study, we show unequivocally that the preconception of FFPE data being noisy, uninterpretable and meaningless is wrong. Our work has shown that, with the described methods, robust signatures can be generated from archival FFPE tumour samples. These can be applied to independent data sets, identify novel biology, and be used to design assays for independent validation with an alternative platform that has potential for clinical exploitation. In conclusion, clinically meaningful and biologically relevant gene expression profiles can be derived from archival FFPE samples with Exon array profiling and this will undoubtedly have significant impact on clinical research and, with time, practice.
