# Supplementary material for: Exon-array profiling unlocks clinically and biologically relevant gene signatures from formalin-fixed paraffin-embedded tumour samples
Source: Br J Cancer. 2011 Mar 15;104(6):971–81. doi: 10.1038/bjc.2011.66 (PMC3065290; doi:10.1038/bjc.2011.66)
Supplement: Supplementary Material [file bjc201166x1.doc]

**Supplementary Material**

**Exon array profiling unlocks clinically and biologically relevant gene signatures from Formalin-Fixed Paraffin-Embedded tumour samples**

John S. Hall, Hui Sun Leong, Lucile S.C. Armenoult, Gillian E. Newton, Helen R. Valentine, Joely J. Irlam, Carla Moller-Levet, Kanwal A. Sikand, Stuart D. Pepper, Crispin J. Miller, Catharine M.L. West.

Contents

Supplementary Methods

- Microarray data analysis pipeline
- p63 immunohistochemistry
- FFPE qRT-PCR

Supplementary Discussions

- Identification of potentially co-regulated novel genes
- Literature-based text-mining analysis using PAKORA

Supplementary Figures

- Figures S1 – S4

Supplementary Tables

- Tables S1 – S5

Supplementary Methods

Microarray data analysis pipeline

*Data preprocessing and probeset filtering*

We performed background adjustment, quantile normalisation and summarisation of probe-level intensity using the Robust Multi-array Average (RMA) algorithm (Irizarry et al., 2003). To remove non-specific and unreliable probesets, two rounds of probeset filtering were carried out. First, a specificity filter was used to keep probesets that were predicted to target a single exonic locus on the genome. Only probesets with all four probes mapping to the target exon were retained at this stage. Annotations and cross-mappings between probesets, exons and genes were obtained from Ensembl human genome build 58 using the R/BioConductor package xmapcore. Exonic probesets that passed the specificity filter were subsequently filtered by Affymetrix detection above background (DABG) scores to remove probesets with signal intensities substantially below the background. The DABG score is an empirical *p*-value derived by comparing the probe intensity to the distribution of the background probe intensities with the same GC content (Affymetrix, 2005). A probeset is called ‘present’ if its DABG *p*-value is less than 0.01. In this analysis, probesets that were flagged as present in at least 3 samples (~10% of all samples) were retained for further analysis. After these filtering steps, 201,102 probesets (representing 30,011 unique genes) remained for differential gene expression analysis.

Identification of probesets differentially expressed between SCC and AC

The R/BioConductor package LIMMA (Smyth, 2004) was used to identify probesets that were differentially expressed between SCC and AC subtypes. We used the method of Benjamini and Hochberg (Benjamini & Hochberg, 1995) to estimate the false discovery rate (FDR) and correct for multiple hypotheses testing. Probesets that changed by at least 2-fold between SCC and AC and with a FDR < 0.01 were considered significant.

Jackknife analysis

We used a resampling technique to assess the stability of the list of differentially expressed probesets obtained in our microarray analysis with respect to perturbations in the original dataset. Using the GeneSelector package in R/BioConductor (Boulesteix & Slawski, 2009), we generated jackknifed datasets for which *k* = 10% and 30% of the samples had been removed from the original dataset. 100 jackknifed datasets were produced for each *k*. In each jackknifed dataset, at least 3 samples from each histology subtype (SCC or AC) were maintained. Then LIMMA gene lists obtained from these jackknifed datasets were compared.

In silico validation of SCC and AC gene signature

We validated the microarray results using an independent cohort reported in Kuner et al. (Kuner et al., 2009). This dataset consists of 58 samples of fresh-frozen human non-small cell lung cancer (NSCLC), of which 40 were classified as AC and 18 as SCC by the authors. The CEL files were downloaded from the Gene Expression Omnibus (GEO) database (accession number GSE10245) and RMA-normalised gene expression values generated. The NSCLC dataset was generated using Affymetrix HG-U133 Plus 2.0 arrays, therefore differentially expressed Exon 1.0 ST probesets were mapped to their corresponding HG-U133 Plus 2.0 probesets prior to the cross-validation analysis. In order to obtain an unambiguous mapping between the two platforms, probesets were mapped to the same genomic loci rather than conversion via the gene symbol. This mapping was performed using the R/BioConductor software xmapcore and the X:Map database (Yates et al., 2008). A two-step strategy was used: 1) identify the genomic coordinates that a specific Exon 1.0 ST probeset located based on genome annotations in Ensembl (build 58), and 2) determine which U133 Plus 2.0 probeset is located within this genomic region. This two-step approach generated a list of 730 U133 Plus 2.0 probesets that are contained within or overlapping the exonic regions spanned by the Exon array probes. These were then subjected to supervised clustering analysis.

Correlation analysis

Correlation analysis was performed to identify genes highly correlated and anti-correlated with *TP63*. First, mean expression values were calculated for each of the 30,011 genes based on their corresponding probesets. Only probesets that passed the probeset filtering criteria were used. Then, pairwise Pearson correlation coefficients between *TP63* and all other genes across the 28 FFPE samples were computed. The top 20 genes that were positively and anti-correlated with *TP63* were identified.

Gene set enrichment analysis

To determine if particular transcription factor binding sites are significantly associated with the histological subtypes SCC and AC, we performed gene set analysis on the transcription factor target gene sets from the Molecular Signature Database (MSigDB version 2.5) using the GSA package in R (Efron & Tibshirani, 2007). The 201,102 probesets that passed our probeset filtering criteria described above were collapsed to unique gene symbols. A mean expression value was calculated for each gene. The analysis was performed using the default settings in GSA except that the maximum gene set size was fixed at 500, and the minimum size set at 15 genes. 1000 permutations were carried out and gene sets satisfying the multiple hypotheses testing threshold FDR ≤ 0.05 were considered as significant.

Identification of over-represented abstract terms using PAKORA

The text-based over-representation analysis tool PAKORA (http://www.pakora.cf.ac.uk/pakora.php) was used to identify PubMed abstract terms associated with the SCC and AC gene lists. The Extended Outlier Detection approach was used to identify significant terms with Bonferroni-adjusted *p*-value < 0.05 as the cutoff.

Ingenuity Pathway Analysis

Ingenuity Pathway Analysis (IPA; Ingenuity Systems, Inc) was performed using v8.7 to map 1062 SCC and 155 AC associated genes using gene symbol. Default parameters were used throughout. For network analysis a 70–member size was considered. The ‘Path Designer’ tool was used to add GSEA motif information and regulated genes, otherwise networks displayed are the highest scoring networks defined by IPA. Tabulated data display only the top Bio-Function categories and canonical pathways identified not all significant categories. All details displayed were significant after multiple testing corrections (Ingenuity).

p63 immunohistochemistry

Sections (4 µm) were dewaxed, rehydrated and the antigen retrieved by microwaving in 10

mM sodium citrate (pH 6.0) for 25 minutes. After quenching endogenous peroxidise, non-specific binding was blocked using 10% casein (Vector Laboratories Ltd., Peterborough, UK). The primary antibody, mouse monoclonal [4A4] – ChiP Grade (Abcam, Cambridge, UK), was applied at 0.2 µg/ml and the sections incubated at 4°C in a humidified chamber overnight. The same concentration of IgG1 control reagent (Dako Ltd., Ely, UK) was used as a negative control. The antigen was detected with Mouse EnVisionPlus reagent (Dako, UK) and visualised with 3,3′-diaminobenzidine (Dako). Sections were then counterstained with haematoxylin, dehydrated and coverslipped. Batch-to-batch variation was assessed by running sections showing high and low p63 expression with each batch.

FFPE qRT-PCR

RNA was reverse transcribed using NuGEN reverse transcription, as per the microarray protocol (NuGEN WT-Ovation systems, Pico). The qRT-PCR was performed using 12 ng cDNA per well and a reaction mix composed of Taqman gene expression Master Mix (Applied Biosystems), primers (0.5 µM) and associated probe (0.25 µM). Each assay was made with a 384 well-plate in triplicate or duplicate. The run was performed on a 7900 thermocycler (Applied Biosystems) and Ct values were obtained using SDS 2.1. Data were normalised to the geometric mean of 4 housekeeping genes, previously validated for use in cervix FFPE samples (*TBP, GAPDH, PGK1, YWHAZ*). Relative values were obtained using the 2^-ΔΔCt^ method. Universal probe library primers and probes were as follows:

*TP63* 5’-gccctcactcctacaaccat-3’ 5’-ggtggggctgagtccatt-3’ #88

*HNF1B* 5’-agaggtccctgcttacctgac-3’ 5’-gcctcctgagagtggattgt-3’ #18

*HNF4A* 5’-cctttctcctccaacccaac-3’ 5’-cagggatcctcacccaagta-3’ #88

Supplementary Discussions

Identification of potentially co-regulated novel genes

Correlation analysis of microarray data is a powerful approach for identifying potentially novel genes involved in biological processes. Given the role of *TP63* as a marker of squamous cells and SCC, we explored which genes correlated with *TP63* in our dataset (potential novel markers of SCC) and which anti-correlated (putative biomarkers for AC). Figure 3 shows a cluster dendrogram of the top 20 genes correlated with *TP63* and the top 20 anti-correlated genes (Pearson correlation). All genes were significant in the LIMMA analysis except for *UTS2R*, which changed by less than 2-fold between SCC and AC. The genes displayed in Figure 3 clearly separate the data into AC and SCC, as expected.

In terms of *TP63* correlated genes, of note are *DSG3*, *DSC3*, *KRT5*, *KRT16*, *KRT6A*, *S100A2* and *S100A8*, which are already described markers of squamous cells. *GPR87* has recently been described as a SCC marker in lung cancer (Gugger et al., 2008). Of particular interest in terms of identifying novel genes involved in squamous differentiation are *DSC3* and *CTA-55I10.1*, a newly annotated transcript with no ascribed function. These two genes had the highest correlation with *TP63* (R^2^=0.954 for both). The genes anti-correlating with *TP63* included *TFF3* and *MUC13* with *EPS8L3* having the highest inverse correlation (R^2^=-0.918). Other genes of note are the cell surface marker *TSPAN8* and 3 genes with unknown function: *RP11-414P19.1*, *AC105391.3* and *C11orf9*. hsa-miR-205 has previously been validated as a clinical biomarker discriminating between SCC and AC was also differentially expressed. This is positively correlated with *TP63* (R^2^=0.66), but falls outside the top 20 positively correlated genes. In terms of deriving a highly specific novel marker gene; *CTA-55I10.1*, *DSC3*, *TRIM29* and *GPR87* are in the top 20 correlated genes for both *TP63* and hsa-mIR-205. Conversely *SPINK1*, *MUC13*, *AC105391.3*, *TSPAN8* and *EPS8L3* are anti-correlated with both *TP63* and hsa-miR-205 (Figure 3 and Supplementary Figure S3).

Literature-based text-mining analysis using PAKORA

To explore the literature further we used text-mining and over-representation analysis. Using PAKORA (Leong & Kipling, 2009) we asked the question which terms in published abstracts were significantly associated with our SCC and AC gene lists (Table S3). The top terms over-represented in the SCC list were keratinocyte, suprabasal, epidermy, cytoskeleton and squamous. Conversely the terms associated with the AC list were epithelia, serous, mucin, epithelial and Calu-3 (a lung cancer adenocarcinoma cell line). This demonstrates that our signature contains genes that are associated with relevant histology from the literature. Both lists represent not only functional differentiation terms, but each list is specifically enriched for epidermoid or glandular terms.

Supplementary Figures


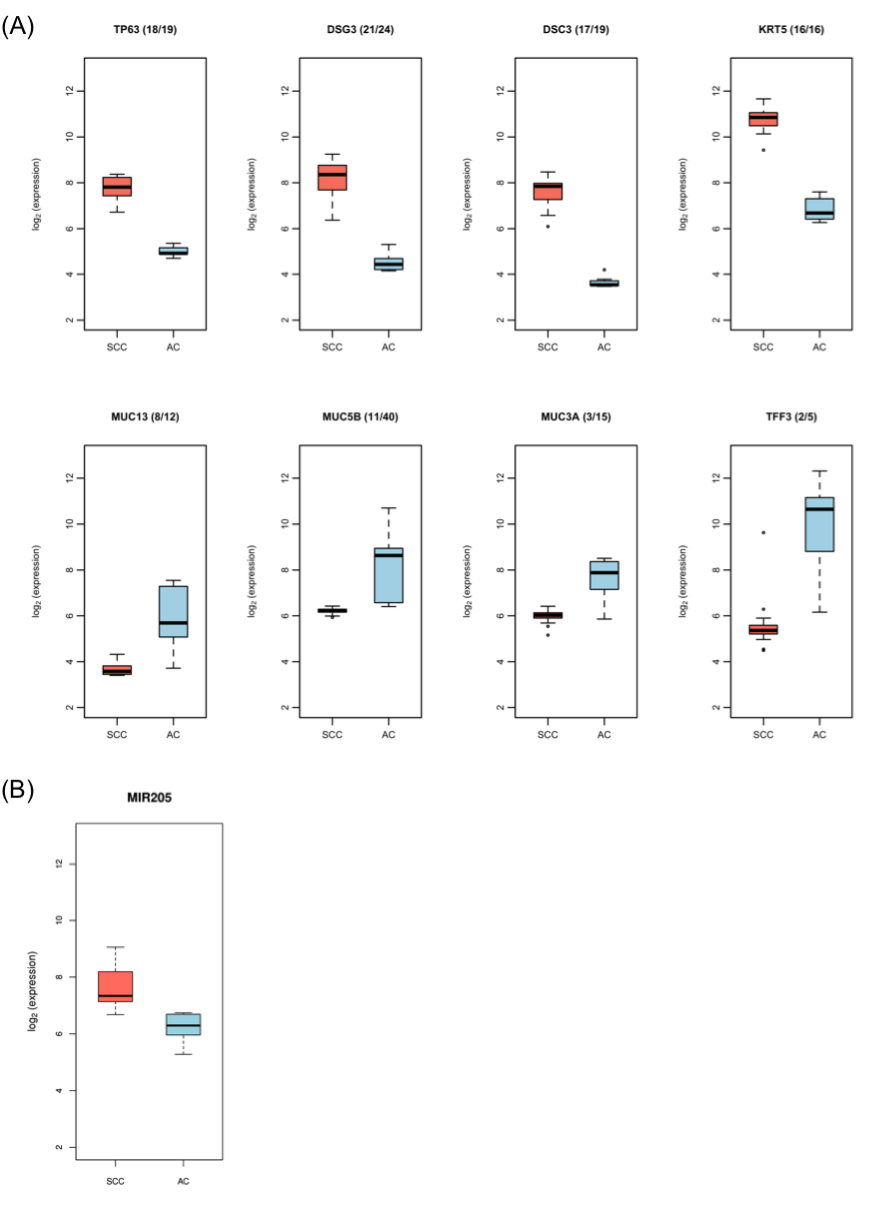
Supplementary Figure S1. Exon array data confirms genes associated with SCC and AC from the literature. A) Literature-based validation of 4 SCC and 4 AC genes. Data shown are derived from 19 SCC and 9 AC samples. B) hsa-miR-205

Supplementary Figure S2. qRT-PCR validation of a subset of differentially expressed genes. A) Roche UPL qRT-PCR for 4 SCC genes and 4AC genes identified by LIMMA across 6 SCC samples and 7 AC samples. Samples are clustered by sample and gene using Euclidean distance. Primers information is listed below the heatmap. B) Primer information.

**
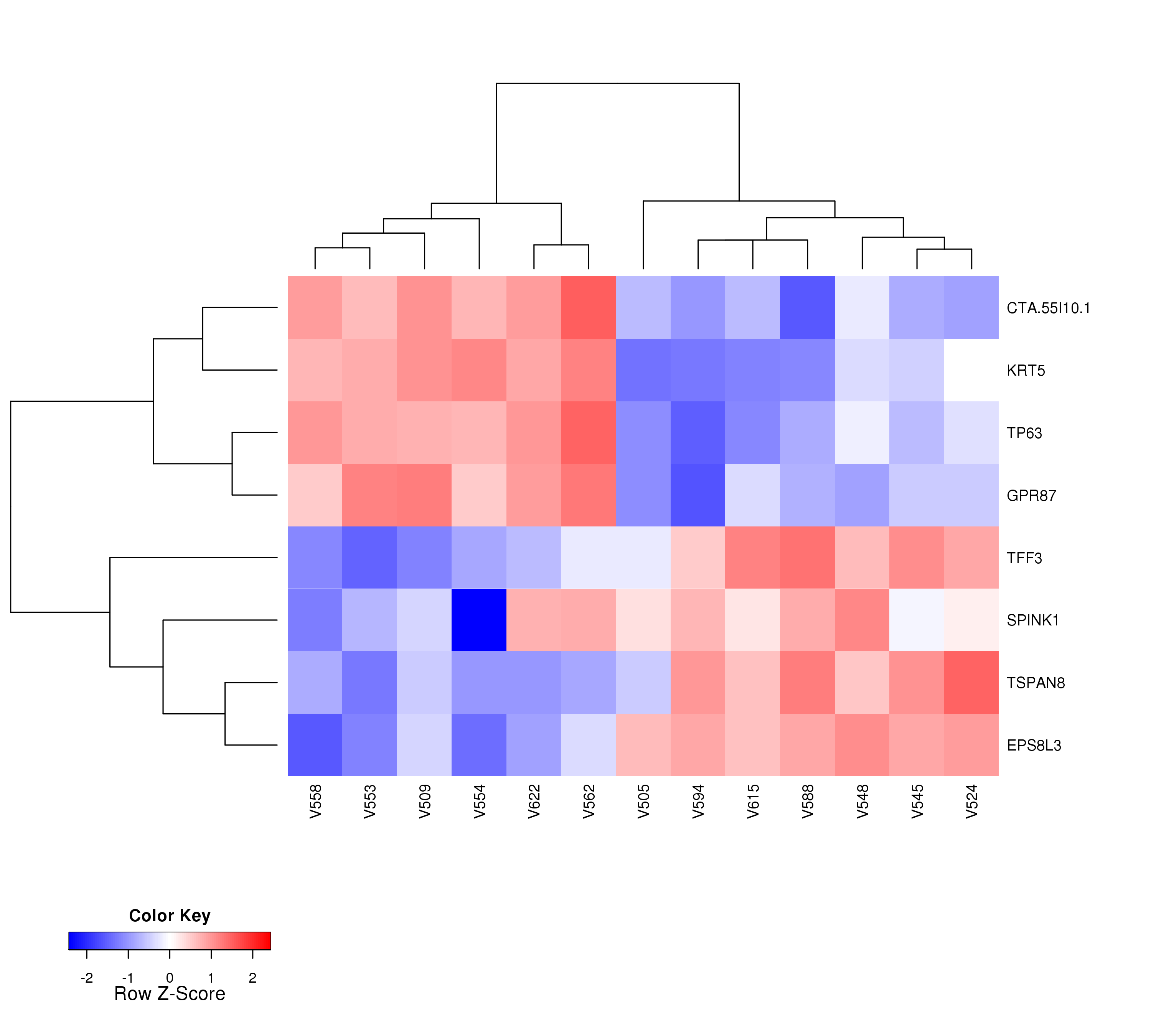
**

**B)**

| **Gene** | **Sense Primer** | **Antisense Primer** | **UPL Probe** | **Amplicon (bp)** |
| --- | --- | --- | --- | --- |
| YWHAZ | cgttacttggctgaggttgc | tgcttgttgtgactgatcgac | 9 | 66 |
| TBP | ctttgcagtgacccagcat | cgctggaactcgtctcacta | 67 | 127 |
| GAPDH | agccacatcgctcagacac | gcccaatacgaccaaatcc | 60 | 66 |
| PGK1 | ctgtggcttctggcatacct | cttgctgctttcaggacca | 42 | 62 |
| SPINK1 | taagtgcggtgcagttttca | tgagaagaaagatgcctgttacc | 41 | 76 |
| EPS8L3 | ggaccacctgctccaagac | cttgacatgttgacgctgct | 79 | 96 |
| TSPAN8 | gtcgctgcatgcttctgtt | taggatacctgtcgccacct | 68 | 74 |
| TFF3 | aagcgcttctgctgaaagtt | agcatgggacctttattcgtt | 50 | 63 |
| GPR87 | caccgtatgaggtgaatgga | tgggttcagcataggttattcc | 83 | 75 |
| KRT5 | tggagaaggagttggaccag | gccatagccactgccact | 6 | 82 |
| CTA-55I10.1 | ttcgtgctctttggacagtg | cagctactctgagcttgcttacc | 55 | 86 |
| TP63 | gccctcactcctacaaccat | ggtggggctgagtccatt | 19 | 102 |

Supplementary Figure S3. Genes that correlate and anti-correlate with hsa-miR-205. Hierarchical clustering of genes and samples was based on Pearson correlation.


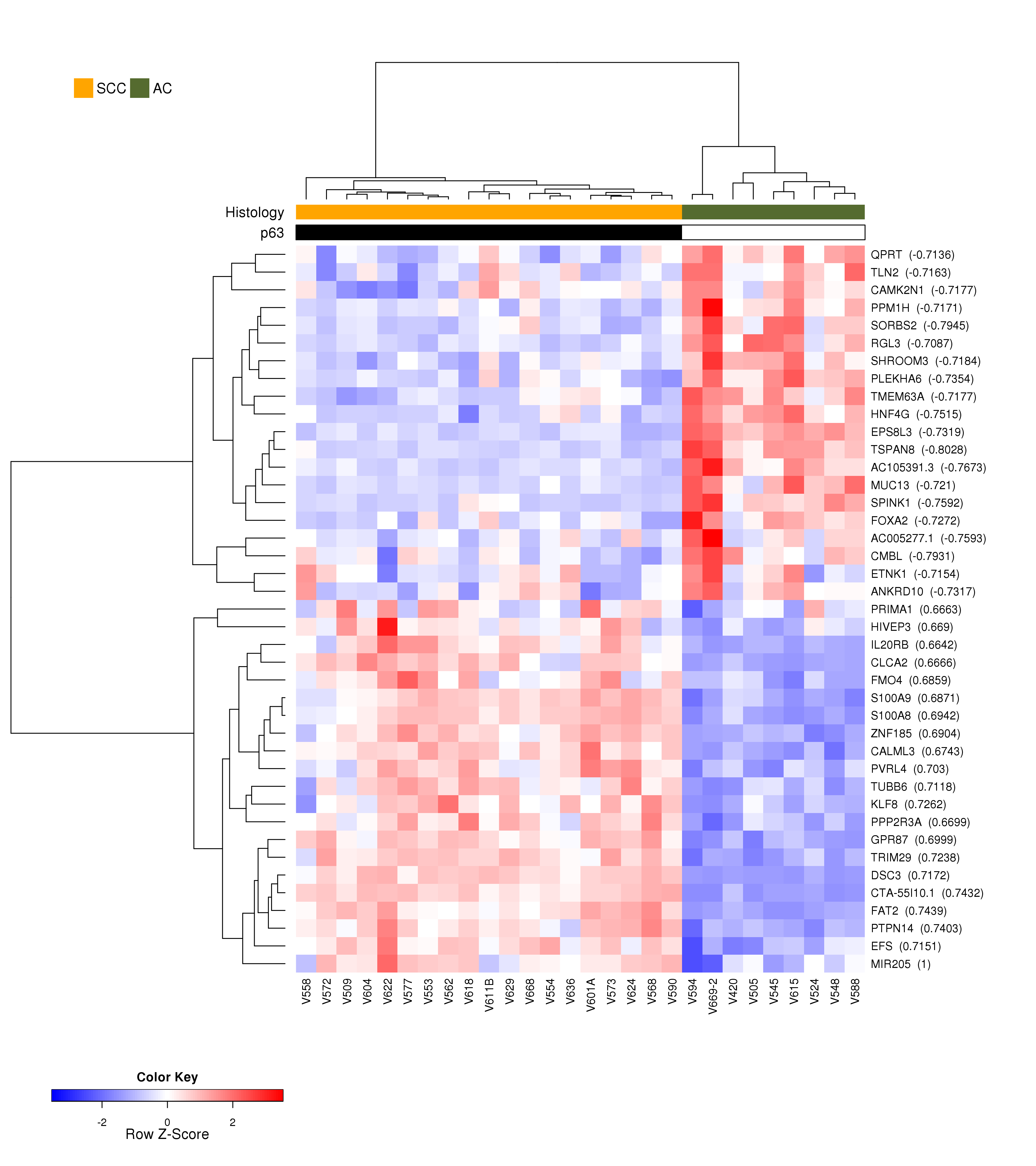


Supplementary Figure S4. Validation of CTA-55I10.1 in cervical carcinoma cell lines. qRT-PCR data showing CTA-55I10.1 and TP63 expression in 16 cervix cancer cell lines. Data shows the mean of a technical triplicate normalised to 4 housekeeping genes.

Supplementary Tables

Supplementary Table S1. Overview of patient demographics

| **Cervix tumour subtype** | | **Squamous Cell Carcinoma (n=19)** | **Adenocarcinoma**  **(n=9)** |
| --- | --- | --- | --- |
| **Parameter** | **Character** |  | |
| Stage | 1b | 1 (5.3%) | 0 (0%) |
|  | 2a-b | 7 (36.8%) | 5 (55.6%) |
|  | 3a-b | 9 (47.3%) | 1 (11.1%) |
|  | 4a-b | 2 (10.5%) | 3 (33.3%) |
|  |  |  |  |
| Patient Age | Median | 63 years [35-81] | 51 years [43-71] |
|  |  |  |  |
| Age of FFPE sample | Median | 11 years [10-13] | 12 years [10-16] |
|  |  |  |  |
| TP63 IHC | Positive | 19 (100%) | 0 (0%) |

[ ] Range ; IHC=immunohistochemistry

Supplementary Table S2. Top ranking differentially expressed genes in cervical squamous cell carcinoma and adenocarcinoma

Top 30 squamous cell carcinoma-specific genes

| **Gene symbol** | **Gene name** | **Ensembl Gene ID** | **Exonic probesets per gene** | **^a^Fraction of DE probesets** | **log2-fold change (SCC/AC)** |
| --- | --- | --- | --- | --- | --- |
| KRT5 | keratin 5 | ENSG00000186081 | 16 | 16/16 | 3.99 |
| DSC3 | desmocollin 3 | ENSG00000134762 | 25 | 17/19 | 3.98 |
| TP63 | tumor protein p63 | ENSG00000073282 | 26 | 18/19 | 2.77 |
| KRT16 | keratin 16 | ENSG00000186832 | 10 | 6/7 | 3.21 |
| CTA-55I10.1 | n/a | ENSG00000230937 | 19 | 16/18 | 3.59 |
| DSG3 | desmoglein 3 (pemphigus vulgaris antigen) | ENSG00000134757 | 26 | 21/24 | 3.65 |
| SFN | stratifin | ENSG00000175793 | 5 | 4/5 | 2.45 |
| S100A8 | S100 calcium binding protein A8 | ENSG00000143546 | 9 | 4/8 | 4.50 |
| FAT2 | FAT tumor suppressor homolog 2 (Drosophila) | ENSG00000086570 | 38 | 21/30 | 2.56 |
| CSTA | cystatin A (stefin A) | ENSG00000121552 | 7 | 5/7 | 3.21 |
| FABP5L4 | fatty acid binding protein 5-like 4 | ENSG00000229287 | 2 | 1/2 | 1.93 |
| S100A2 | S100 calcium binding protein A2 | ENSG00000196754 | 16 | 8/12 | 3.47 |
| IL20RB | interleukin 20 receptor beta | ENSG00000174564 | 19 | 5/13 | 2.59 |
| TRIM29 | tripartite motif-containing 29 | ENSG00000137699 | 19 | 10/15 | 2.30 |
| CLCA2 | chloride channel accessory 2 | ENSG00000137975 | 28 | 19/24 | 2.98 |
| KRT6A | keratin 6A | ENSG00000205420 | 2 | 2/2 | 4.37 |
| DENND2C | DENN/MADD domain containing 2C | ENSG00000175984 | 33 | 11/23 | 2.01 |
| PKP1 | plakophilin 1 (ectodermal dysplasia/skin fragility syndrome) | ENSG00000081277 | 22 | 9/18 | 1.76 |
| DSC2 | desmocollin 2 | ENSG00000134755 | 27 | 19/19 | 2.78 |
| ZNF185 | zinc finger protein 185 (LIM domain) | ENSG00000147394 | 28 | 10/20 | 2.02 |
| S100A9 | S100 calcium binding protein A9 | ENSG00000163220 | 6 | 4/5 | 3.48 |
| SERPINB13 | serpin peptidase inhibitor, clade B (ovalbumin), member 13 | ENSG00000197641 | 14 | 10/13 | 2.70 |
| A2ML1 | alpha-2-macroglobulin-like 1 | ENSG00000166535 | 42 | 19/34 | 2.66 |
| MTSS1 | metastasis suppressor 1 | ENSG00000170873 | 29 | 13/23 | 2.11 |
| TMEM40 | transmembrane protein 40 | ENSG00000088726 | 18 | 1/11 | 2.48 |
| TFAP2A | transcription factor AP-2 alpha (activating enhancer binding protein 2 alpha) | ENSG00000137203 | 35 | 8/26 | 2.07 |
| LYPD3 | LY6/PLAUR domain containing 3 | ENSG00000124466 | 10 | 2/9 | 2.21 |
| RNF19B | ring finger protein 19B | ENSG00000116514 | 18 | 4/13 | 1.88 |
| GPR87 | G protein-coupled receptor 87 | ENSG00000138271 | 9 | 7/9 | 2.87 |
| CALML3 | calmodulin-like 3 | ENSG00000178363 | 4 | 2/4 | 2.46 |

**Supplementary Table S2** (continued)

Top 30 adenocarcinoma-specific genes

| **Gene symbol** | **Gene name** | **Ensembl Gene ID** | **Exonic probesets per gene** | **Fraction of DE probesets** | **log2-fold change (SCC/AC)** |
| --- | --- | --- | --- | --- | --- |
| EPS8L3 | EPS8-like 3 | ENSG00000198758 | 29 | 3/17 | -2.91 |
| KIF12 | kinesin family member 12 | ENSG00000136883 | 31 | 2/11 | -1.85 |
| TSPAN8 | tetraspanin 8 | ENSG00000127324 | 17 | 7/12 | -3.51 |
| USH1C | Usher syndrome 1C (autosomal recessive, severe) | ENSG00000006611 | 33 | 7/15 | -2.84 |
| HGD | homogentisate 1,2-dioxygenase (homogentisate oxidase) | ENSG00000113924 | 48 | 2/23 | -4.89 |
| SPINK1 | serine peptidase inhibitor, Kazal type 1 | ENSG00000164266 | 7 | 4/4 | -3.40 |
| TFF3 | trefoil factor 3 (intestinal) | ENSG00000160180 | 9 | 2/5 | -4.46 |
| SLC44A4 | choline transporter-like protein 4 | ENSG00000204385 | 25 | 5/18 | -2.11 |
| RP11-414P19.1 | n/a | ENSG00000235413 | 4 | 1/2 | -2.04 |
| ALDH1L2 | aldehyde dehydrogenase 1 family, member L2 | ENSG00000136010 | 24 | 1/13 | -2.55 |
| C2orf28 | Apoptosis-related protein 3 Precursor (APR-3)(p18) | ENSG00000138085 | 14 | 1/10 | -1.21 |
| AC105391.3 | Putative Rho guanine nucleotide exchange factor FLJ20184 | ENSG00000236699 | 24 | 5/11 | -1.96 |
| UPB1 | ureidopropionase, beta | ENSG00000100024 | 21 | 1/6 | -1.34 |
| GAL3ST1 | galactose-3-O-sulfotransferase 1 | ENSG00000128242 | 12 | 1/4 | -3.08 |
| PROM1 | prominin 1 | ENSG00000007062 | 36 | 14/23 | -2.22 |
| RGL3 | ral guanine nucleotide dissociation stimulator-like 3 | ENSG00000205517 | 24 | 2/8 | -2.16 |
| PABPC1P2 | poly(A) binding protein, cytoplasmic 1 pseudogene 2 | ENSG00000198526 | 16 | 1/4 | -1.16 |
| CCDC114 | coiled-coil domain containing 114 | ENSG00000105479 | 27 | 1/7 | -1.94 |
| CLDN3 | claudin 3 | ENSG00000165215 | 5 | 1/2 | -1.63 |
| PDPN | podoplanin | ENSG00000162493 | 12 | 1/8 | -1.31 |
| DDAH1 | dimethylarginine dimethylaminohydrolase 1 | ENSG00000153904 | 25 | 3/11 | -2.04 |
| SLC6A19 | solute carrier family 6 (neutral amino acid transporter), member 19 | ENSG00000174358 | 20 | 1/4 | -1.20 |
| TSPAN12 | tetraspanin 12 | ENSG00000106025 | 18 | 2/9 | -1.71 |
| CLRN3 | clarin 3 | ENSG00000180745 | 6 | 1/2 | -2.29 |
| PPP1R9A | protein phosphatase 1, regulatory (inhibitor) subunit 9A | ENSG00000158528 | 36 | 2/21 | -2.16 |
| PTGES2 | prostaglandin E synthase 2 | ENSG00000148334 | 22 | 1/14 | -1.05 |
| GJB1 | gap junction protein, beta 1, 32kDa | ENSG00000169562 | 9 | 2/5 | -1.42 |
| EFCAB4A | EF-hand calcium binding domain 4A | ENSG00000177685 | 17 | 1/7 | -1.16 |
| ANKS4B | ankyrin repeat and sterile alpha motif domain containing 4B | ENSG00000175311 | 6 | 1/1 | -2.16 |
| AGR2 | anterior gradient homolog 2 (Xenopus laevis) | ENSG00000106541 | 14 | 6/9 | -2.35 |

^a^ Fraction of DE probeset: This is the number of significant probeset over the total analysed in LIMMA. The denominator is the number of exonic probesets associated with a particular gene, which are considered as uniquely-mapped and pass the DABG-filtering criteria described in the text.

Supplementary Table S3. Text-based over-representation analysis results

Top 10 PubMed abstract terms significantly associated with the 1062 SCC genes

| **Term** | **List** | **Z-score** | **Raw P-value** | **Corrected P-value** | **Ranking** |
| --- | --- | --- | --- | --- | --- |
| Keratinocyte | 99 | 7.56 | 1.98E-14 | 2.02E-10 | 1 |
| Suprabasal | 31 | 6.64 | 1.52E-11 | 1.55E-07 | 2 |
| Epidermy | 66 | 6.49 | 4.18E-11 | 4.27E-07 | 3 |
| Cytoskeleton | 90 | 5.34 | 4.62E-08 | 0.000471 | 4 |
| Squamous | 87 | 5.29 | 6.26E-08 | 0.000639 | 5 |
| Epithelia | 74 | 4.94 | 3.92E-07 | 0.004 | 6 |
| Microtubule | 73 | 4.88 | 5.40E-07 | 0.00551 | 7 |
| Desmosomal | 15 | 4.74 | 1.07E-06 | 0.0109 | 8 |
| Nail | 17 | 4.71 | 1.27E-06 | 0.0129 | 9 |
| Downregulate | 89 | 4.67 | 1.50E-06 | 0.0153 | 10 |

Top 10 PubMed abstract terms significantly associated with the 155 AC genes

| **Term** | **List** | **Z-score** | **Raw P-value** | **Corrected P-value** | **Ranking** |
| --- | --- | --- | --- | --- | --- |
| Epithelia | 25 | 7.21 | 2.70E-13 | 8.94E-10 | 1 |
| Serous | 13 | 6.51 | 3.70E-11 | 1.23E-07 | 2 |
| Mucin | 12 | 6.1 | 5.41E-10 | 1.79E-06 | 3 |
| Epithelial | 48 | 5.97 | 1.20E-09 | 3.99E-06 | 4 |
| Calu-3 | 5 | 5.88 | 2.05E-09 | 6.80E-06 | 5 |
| Duct | 18 | 5.77 | 4.04E-09 | 1.34E-05 | 6 |
| Mucus | 8 | 5.23 | 8.53E-08 | 0.000283 | 7 |
| Goblet | 7 | 5.17 | 1.14E-07 | 0.000379 | 8 |
| Muc5ac | 5 | 4.96 | 3.49E-07 | 0.00116 | 9 |
| Intestinal | 22 | 4.92 | 4.44E-07 | 0.00147 | 10 |

Supplementary Table S4. Biological function analysis of SCC and AC genes

Top SCC biological functions from Ingenuity

| Category | *P*-value | Size | Examples |
| --- | --- | --- | --- |
| **Disease and Disorders** | | | |
| Dermatological Diseases and Conditions | 8.50E-08 | 85 | KRT5, TP63, KRT16, DSG3, S100A8, KRT6A, PKP1, S100A9, DSP, ZNF750 |
| Cancer | 1.36E-07 | 291 | KRT5, TP63, KRT16, DSG3, SFN, S100A8, S100A2, TRIM29, KRT6A, ZNF185 |
| Genetic Disorder | 1.09E-05 | 498 | KRT5, DSC3, TP63, KRT16, DSG3, S100A8, S100A2, TRIM29, KRT6A, PKP1 |
| Respiratory Disease | 1.09E-05 | 50 | KRT5, TP63, DSG3, THBD, MAFB, CD44, KRT6B, CYP19A1, EGFR, CHI3L1 |
| Reproductive System Disease | 5.41E-05 | 127 | KRT5, TP63, S100A8, S100A2, KRT6A, S100A9, SERPINB5, CD44, MICALL1, MAF |
| **Molecular and Cellular Function** | | | |
| Cell Death | 1.08E-11 | 254 | TP63, DSG3, SFN, S100A8, CSTA, S100A9, TFAP2A, DSP, DUSP7, THBD |
| Cellular Development | 6.93E-10 | 185 | TP63, SFN, CSTA, KRT6A, TFAP2A, DSP, THBD, PITX1, MAFB, IFI16 |
| Cellular Function and Maintenance | 7.50E-08 | 98 | TP63, S100A9, MTSS1, TFAP2A, IFI16, CD44, TIAM1, TNFSF10, MYO1E, DST |
| Cell Morphology | 9.25E-08 | 126 | TP63, KRT16, MTSS1, TFAP2A, CALML3, SERPINB5, CD44, TIAM1, IRF6, PVRL1 |
| Cellular Movement | 1.13E-07 | 165 | TP63, KRT16, S100A8, S100A2, KRT6A, S100A9, MTSS1, TFAP2A, LYPD3, CALML3 |
| **Physiological System Development and Function** | | | |
| Hair and Skin Development and Function | 2.00E-09 | 55 | KRT5, TP63, KRT16, DSG3, SFN, CSTA, IL20RB, DSP, KRT15, TIAM1 |
| Organ Development | 2.00E-09 | 32 | KRT5, TP63, KRT16, SFN, DSP, KRT15, CALML5, IRF6, CLDN1, SPRR1A |
| Tissue Development | 6.55E-07 | 98 | DSC3, KRT16, DSG3, S100A8, DSC2, S100A9, THBD, PITX1, SERPINB5, CSTB |
| Embryonic Development | 1.47E-05 | 39 | SERPINB5, IL1RN, CD44, TNFSF10, FOSL2, HSPB1, CD9, PRMT2, EGFR, IPPK |
| Cardiovascular System Development and Function | 2.40E-05 | 37 | SERPINB5, IFI16, CDKN2B, STX6, RAB10, TNFSF10, EGFR, NCK1, TUBB6, TUBA4A |
| **Canonical Pathways** | | | |
| Breast Cancer Regulation by Stathmin1 | 2.45E-06 | 28 | PPP2R3A, CALML5, PRKCH, ADCY3, ARHGEF4, PIK3C3, GNAI1, CALM1, TUBA4A, CDKN1A |
| p70S6K Signaling | 2.24E-05 | 20 | SFN, PPP2R3A, PRKCH, EGFR, PIK3C3, GNAI1, PLD1, PPP2R2A, MTOR, PPP2R5E |
| Rac Signaling | 9.55E-05 | 17 | CD44, TIAM1, PRKCH, ARPC2, PIK3C3, PLD1, PARD3, PAK2, ITGA2, IQGAP1 |
| Regulation of eIF4 and p70S6K Signaling | 1.23E-04 | 17 | PPP2R3A, PIK3C3, EIF4G2, PPP2R2A, ITGA2, MTOR, PPP2R5E, SOS2, RAF1, EIF2A |
| PI3K/AKT Signaling | 1.51E-04 | 18 | SFN, PPP2R3A, GSK3B, CDKN1A, PPP2R2A, ITGA2, MTOR, NFKBIA, PPP2R5E, SOS2 |
| p53 Signaling | 0.000224 | 15 | TP63, SFN, SERPINB5, GSK3B, PERP, PIK3C3, SNAI2, CDKN1A, PRKDC, PMAIP1 |
| fMLP Signaling in Neutrophils | 0.000389 | 16 | CALML5, PRKCH, ARPC2, PIK3C3, GNAI1, CALM1, GNG12, NFKBIA, ARPC5, RAF1 |
| Glioma Signaling | 0.000407 | 15 | CDKN2B, CALML5, PRKCH, EGFR, PIK3C3, CALM1, RBL1, CDKN1A, MTOR, SOS2 |
| HER-2 Signaling in Breast Cancer | 0.000407 | 13 | PRKCH, PARD6G, EGFR, GSK3B, PIK3C3, PARD3, CDKN1A, SOS2, HRAS, RAC1 |
| Molecular Mechanisms of Cancer | 0.000468 | 35 | CDKN2B, FZD6, GNA15, RAP2B, PRKCH, ADCY3, GSK3B, ARHGEF4, PIK3C3, GNAI1 |

**Supplementary Table S4** (continued)

Top AC biological functions from Ingenuity

| Category | *P*-value | Size | Examples |
| --- | --- | --- | --- |
| **Disease and Disorders** | | | |
| Cancer | 7.96E-08 | 57 | SPINK1, TFF3, PROM1, CLDN3, PDPN, TSPAN12, GJB1, AGR2, MUC13, PAX8 |
| Gastrointestinal Disease | 2.78E-07 | 43 | SPINK1, TFF3, PROM1, CLDN3, PDPN, GJB1, MUC13, CDH17, CACNA1D, LGALS4 |
| Genetic Disorder | 4.65E-07 | 87 | TSPAN8, USH1C, HGD, SPINK1, TFF3, UPB1, PROM1, CLDN3, PDPN, DDAH1 |
| Reproductive System Disease | 9.89E-06 | 30 | TFF3, PROM1, CLDN3, PDPN, AGR2, PAX8, TMC5, LGALS4, CHRND, PKHD1 |
| Hepatic System Disease | 0.000648 | 10 | GJB1, CHRND, C3, ABCC3, HNF4A, HNF1B, IRS2, CFTR, SLC12A2, PDE4D |
| **Molecular and Cellular Function** | | | |
| Amino Acid Metabolism | 5.88E-05 | 9 | HGD, UPB1, PDPN, DDAH1, SLC6A19, GLDC, ABCC3, HNF4A, FOLR1 |
| Drug Metabolism | 5.88E-05 | 7 | PDPN, SULT1C2, ABCC3, IRS2, PRKCA, SLC12A2, FOLR1 |
| Molecular Transport | 5.88E-05 | 17 | GAL3ST1, PDPN, SLC6A19, GJB1, CDH17, C3, ABCC3, HNF4A, CXCL3, IRS2 |
| Small Molecule Biochemistry | 5.88E-05 | 29 | HGD, UPB1, GAL3ST1, PDPN, DDAH1, SLC6A19, GJB1, CDH17, SULT1C2, ABP1 |
| Vitamin and Mineral Metabolism | 5.88E-05 | 7 | PDPN, CACNA1D, ABCC3, PRKCA, BCMO1, PDE4D, FOLR1 |
| **Physiological System Development and Function** | | | |
| Tissue Development | 0.000187 | 24 | TFF3, CLDN3, PDPN, CDH17, CACNA1D, LGALS4, PKHD1, EPCAM, C3, CLDN10 |
| Metabolic Disease | 0.000767 | 41 | TSPAN8, USH1C, HGD, SLC44A4, DDAH1, SLC6A19, CLRN3, PAX8, CACNA1D, CHRND |
| Immune Cell Trafficking | 0.000781 | 10 | GAL3ST1, CCL15, C3, CXCL3, PRKCA, CFTR, DMBT1, PIGR, PDE4D, NEDD9 |
| Lymphoid Tissue Structure and Development | 0.000781 | 6 | CCL15, C3, CXCL3, PRKCA, PIGR, PDE4D |
| Endocrine System Disorders | 0.002870 | 38 | TSPAN8, USH1C, HGD, SPINK1, SLC44A4, DDAH1, CLRN3, GJB1, PAX8, CACNA1D |
| **Canonical Pathways** | | | |
| Maturity Onset Diabetes of Young (MODY) Signaling | 0.000708 | 3 | CACNA1D, HNF4A, HNF1B |
| Glycine, Serine and Threonine Metabolism | 0.003020 | 4 | ABP1, GLDC, AGXT2, PLCB4 |
| Aldosterone Signaling in Epithelial Cells | 0.003981 | 4 | PRKCA, SLC12A2, PIP5K1B, PLCB4 |
| Airway Pathology in Chronic Obstructive Pulmonary Disease | 0.063096 | 1 | CXCL3 |
| Leukocyte Extravasation Signaling | 0.064565 | 4 | CLDN3, CLDN10, MMP15, PRKCA |
| Thrombopoietin Signaling | 0.067608 | 2 | IRS2, PRKCA |
| _-alanine Metabolism | 0.067608 | 2 | UPB1, ABP1 |
| Hepatic Cholestasis | 0.087096 | 3 | ABCC3, HNF4A, PRKCA |
| Cellular Effects of Sildenafil (Viagra) | 0.089125 | 3 | CACNA1D, PLCB4, PDE4D |
| PI3K Signaling in B Lymphocytes | 0.095499 | 3 | C3, IRS2, PLCB4 |

Supplementary Table S5. Gene set enrichment analysis

Motif gene sets that are significantly enriched in the SCC samples as identified by using GSEA based on 1000 permutations (FDR < 0.05)

| Gene set name | Description | DEG in gene set |
| --- | --- | --- |
| SMTTTTGT_UNKNOWN | Genes with promoter regions [-2kb,2kb] around transcription start site containing motif SMTTTTGT. Motif does not match any known transcription factor | MTSS1, DAAM1, TIAM1, RAB10, TNFSF10, ARPC2, MSN, MAPK6, JUP, NIN, GSK3B, SLC38A2, ETS2, EIF4G2, LCP1, KLF7, YWHAQ, BCL11A, KIF1B, RCOR1, ZBTB11, STMN1, QKI, BMPR2, CCBL1, EPN2, YWHAG, ITGB4, SRGAP2, IRAK1, AMMECR1, FKBP5, PDE4D, MMP14, ATRX, CPEB4, BCL6, PANK3, CSNK1A1 |

Motif gene sets that are significantly enriched in the AC samples as identified by using GSEA based on 1000 permutations (FDR < 0.05)

| Gene set name | Description | DEG in gene set |
| --- | --- | --- |
| RGTTAMWNATT_V$HNF1_01 | Genes with promoter regions [-2kb,2kb] around transcription start site containing the motif RGTTAMWNATT which matches annotation for TCF1: transcription factor 1, hepatic; LF-B1, hepatic nuclear factor (HNF1), albumin proximal factor | NFE2L2, SGK2, SLC12A2, RTN4, COL16A1 |
| V$HNF1_01 | Genes with promoter regions [-2kb,2kb] around transcription start site containing the motif GGTTAATNWTTAMCN which matches annotation for TCF1: transcription factor 1, hepatic; LF-B1, hepatic nuclear factor (HNF1), albumin proximal factor | KIF12, PVRL1, NFE2L2, CDH17, ZNF148, SLC4A4, PKHD1, SGK2, SEMA4G, HNF4A, TLE4, BCL11A, GAN, GATA6, DPYD |
| V$HNF1_C | Genes with promoter regions [-2kb,2kb] around transcription start site containing the motif DGTTAATKAWTNACCAM which matches annotation for TCF1: transcription factor 1, hepatic; LF-B1, hepatic nuclear factor (HNF1), albumin proximal factor | SPINK1, DAAM1, PVRL1, CAST, MARCKS, GJB1, CDH17, PLS3, PKHD1, SGK2, SEMA4G, MXI1, PRDM1, YES1, TLE4, SLC12A2, C8A, CGN, TRPS1, LRRFIP2, DPYD, RASA2 |
| V$HNF1_Q6 | Genes with promoter regions [-2kb,2kb] around transcription start site containing the motif WRGTTAATNATTAACNNN which matches annotation for TCF1: transcription factor 1, hepatic; LF-B1, hepatic nuclear factor (HNF1), albumin proximal factor | SPINK1, DAAM1, GJB1, CDH17, SLC4A4, PKHD1, SGK2, SEMA4G, MIA2, HOXD10, CLDN10, HNF4A, PRDM1, CXCL3, TLE4, BCL11A, SLC12A2, C8A, GAN, STAG2, LRRFIP2, DPYD, PDE4D |

Supplementary References

Affymetrix. (2005). White Paper, Exon Array Background Correction v1.0.

Benjamini, Y. & Hochberg, Y. (1995). Controlling the false discovery rate: a practical and powerful approach to multiple testing. *Journal of the Royal Statistical Society Series B*, **57,** 289-300.

Boulesteix, A.L. & Slawski, M. (2009). Stability and aggregation of ranked gene lists. *Brief Bioinform*, **10,** 556-68.

Efron, B. & Tibshirani, R. (2007). On testing the significance of sets of genes. . *Ann. Appl. Stat.*, **1,** 107-129.

Gugger, M., White, R., Song, S., Waser, B., Cescato, R., Riviere, P. & Reubi, J.C. (2008). GPR87 is an overexpressed G-protein coupled receptor in squamous cell carcinoma of the lung. *Dis Markers*, **24,** 41-50.

Irizarry, R.A., Hobbs, B., Collin, F., Beazer-Barclay, Y.D., Antonellis, K.J., Scherf, U. & Speed, T.P. (2003). Exploration, normalization, and summaries of high density oligonucleotide array probe level data. *Biostatistics*, **4,** 249-64.

Kuner, R., Muley, T., Meister, M., Ruschhaupt, M., Buness, A., Xu, E.C., Schnabel, P., Warth, A., Poustka, A., Sultmann, H. & Hoffmann, H. (2009). Global gene expression analysis reveals specific patterns of cell junctions in non-small cell lung cancer subtypes. *Lung Cancer*, **63,** 32-8.

Leong, H.S. & Kipling, D. (2009). Text-based over-representation analysis of microarray gene lists with annotation bias. *Nucleic Acids Res*, **37,** e79.

Smyth, G.K. (2004). Linear models and empirical bayes methods for assessing differential expression in microarray experiments. *Stat Appl Genet Mol Biol*, **3,** Article3.

Yates, T., Okoniewski, M.J. & Miller, C.J. (2008). X:Map: annotation and visualization of genome structure for Affymetrix exon array analysis. *Nucleic Acids Res*, **36,** D780-6.
